# Serum Endoglin Levels in Patients Suffering from Systemic Sclerosis and Elevated Systolic Pulmonary Arterial Pressure

**DOI:** 10.1155/2010/969383

**Published:** 2010-08-24

**Authors:** Paola Ximena Coral-Alvarado, Maria Fernanda Garces, Jorge Eduardo Caminos, Antonio Iglesias-Gamarra, José Félix Restrepo, Gerardo Quintana

**Affiliations:** ^1^Rheumatology Section, Fundación Santa Fe de Bogotá, School Medicine Universidad de los Andes, Bogotá, Colombia; ^2^Rheumatology Unit, Universidad Nacional de Colombia, Bogotá, Colombia; ^3^Biochemistry Unit, Universidad Nacional de Colombia, Bogotá, Colombia

## Abstract

*Background*. Pulmonary arterial hypertension (PAH) is the main cause of morbimortality in systemic sclerosis (SSc). Increased Eng expression has been demonstrated in SSc patients. *Objective*. Ascertaining serum levels of Eng in SSc patients with and without elevated systolic pulmonary arterial pressure (sPAP) and comparing them with that of healthy volunteers. *Methods*. A cross-sectional study was carried out. A commercial ELISA kit was used for measuring serum concentrations of Eng in 60 subjects: 40 patients with SSc with and without elevated sPAP, compared to 20 healthy control subjects. Elevated sPAP was detected by echocardiogram. *Results*. No association between positive Eng and elevated sPAP was found when compared to the SSc without elevated sPAP group (OR = 2.85; 0.65–12.88 95% CI; *P* = .11); however, an association was found between positive Eng and elevated sPAP compared to healthy controls (OR = 23.22; 2.46–1050.33 95% CI; *P* = .001), and weak association was found between the positive Eng with SSc without elevated sPAP group compared to healthy controls (OR = 8.14, 0.8–393.74 95% CI; *P* = .046). *Conclusion*. Raised serum levels of Eng in SSc patients compared to healthy controls were found, suggesting a role for Eng in SSc vasculopathy and not just in elevated sPAP. However, prospective studies are needed to verify such observations.

## 1. Introduction

Systemic sclerosis (SSc) is an autoimmune disease having an unknown etiology characterized by microvasculopathy, immunological abnormalities, and excessive collagen deposits [1, 2].Endothelial dysfunction and microcirculation damage are cardinal features of systemic sclerosis (SSc); it is thought that vascular changes occur at an early stage and may include endothelial cell apoptosis, endothelium activation, inflammatory cell recruitment, intimal proliferation, and adventitial fibrosis, all of which may lead to vessel obliteration [3].

Although SSc pathogenesis remains uncertain, increasing evidence suggests that transforming growth factor-beta (TGF-*β*) plays a key role in tissue fibrosis development, a consequence of extracellular matrix accumulation in SSc pathogenesis. TGF-*β* regulates diverse biological activities including cell growth, apoptosis, differentiation, and extracellular matrix synthesis through interaction with TGF-*β* receptors [4, 5].

Endoglin (Eng) is a glycoprotein having antiangiogenic properties that acts as a TGF-*β* receptor complex component. Eng may act on fibroblasts to modulate TGF-*β* signaling by acting as a molecular sink regulating or reducing the total pool of TGF-*β* available for activating signal-transducing receptors.

Increased expression in fibroblasts and endothelial cells has been demonstrated in SSc patients, suggesting that deregulating Eng expression and/or function may be related to the vascular manifestation of SSc [6, 7].

Pulmonary arterial hypertension (PAH) is the main vascular complication in SSc, being an important cause of morbidity and the main cause of mortality, having 50% survival rate at 12 months [8]. This vasculopathy is caused by a number of soluble factors and involves a complex interaction between endothelial cells, smooth muscle cells, extracellular matrix, coagulation factors, and circulating cells [9].

There are two types of PAH in SSc: PAH secondary to fibrosis and severe interstitial involvement and isolated PAH without fibrosis or interstitial lung disease, with the latter reflecting the illness' vascular pathology and resulting in a more indolent pulmonary process [8].

PAH prevalence in SSc varies depending on the methods used for diagnosis. Estimated PAH prevalence is 13.3% when using echocardiogram [10]. Cardiac catheterization is considered the gold standard for PAH diagnosis, but this technique is invasive, expensive, and not available in all centers. It can sometimes represent a high-risk factor for morbidity; this is why its use for detecting PAH and its followup are limited [11]. Markers should thus be sought to enable detecting patient subgroups having the highest risk of developing PAH as a vascular complication of SSc and allowing followup. This would enable PAH to be diagnosed as early as possible, thereby improving prognosis for PAH and SSc patients.

This study examined serum levels of Eng in patients with SSc and elevated sPAP (SSc-sPAP) and SSc without elevated sPAP (SSc-non sPAP) and these were compared with healthy volunteers for determining any association between Eng levels and elevated sPAP in SSc patients.

## 2. Materials and Methods

### 2.1. Patients

A cross-sectional study was carried out between June and October 2008 during which 60 subjects were analyzed; 40 patients met American College of Rheumatology (ACR) criteria [12] for SSc (20 patients had elevated sPAP by echocardiogram and 20 patients did not), and 20 control healthy subjects were also included. The patients were consecutively selected based on the available and willingness to participate in this study. Patients who presented any other connective tissue disease or a concomitant pulmonary illness from any other etiology, work, or environmental exposure for pulmonary disease were excluded.

Registration forms were completed which included demographic data, clinical characteristics, antibody levels, reports from diagnostic tools such as echocardiogram and high-resolution computed tomography of the thorax (HRCTT).

The patients were subdivided into two groups: limited SSc (lSSc) and diffuse SSc (dSSc) based on the limits proposed by LeRoy et al. [1]. Blood samples were drawn from the 40 SSc patients and from 20 healthy subjects matched by age and gender. No patient was receiving calcium channel blockers when being analyzed. All samples were stored at −20°C until being processed. The protocol was approved by the Universidad Nacional de Colombia's ethics committee and all patients signed the informed consent forms agreeing to take part in this study.

### 2.2. Echocardiogram

All echocardiograms were taken by an expert cardiologist using standard techniques for evaluating right ventricle dimensions and tricuspid gradients after a 20-minute rest. The tricuspid systolic pressure gradient was calculated by using Bernoulli's modified equation [13].

#### 2.2.1. Estimating Elevated Systolic Pulmonary Arterial Pressure

The sPAP was calculated as being the sum of the tricuspid gradient and estimated right atrial pressure. Elevated sPAP was defined in Colombian patients living at 2,600 meters above sea level as being mean >35 mmHg sPAP, >3 m/second tricuspid regurgitation velocity, or 2.5 m/second in patients having unexplained dyspnea [14]. Elevated sPAP was defined as > 35 mmHg sPAP with HRCTT without evidence of interstitial lung disease (such as bibasilar pulmonary fibrosis or reticulonodular densities, being most pronounced in the lung bases), or the presence of heterogeneous opacities such as reticular opacities, ground-glass opacities, or honeycombing in HRCTT [14].

### 2.3. ELISA

A commercial ELISA kit (R&D Systems, Minneapolis, Minn, USA) was used for serum measurement for Eng from SSc patients and the control group, following the manufacturer's protocol. Each sample was done twice.

### 2.4. Statistical Analysis

STATA 9.0 software was used for statistical analysis. The Shapiro-Wilks test was used for evaluating data distribution. Clinical data, elevated sPAP and Eng serum values were compared by unpaired Student *t*-test or Mann-Whitney *U* test as appropriate. Fisher's exact test or Chi-^2^test was used for determining association between categorical variables. Odds ratios (OR) with 95% confidence interval (95% CI) were also reported. The Kruskal-Wallis test or ANOVA was used for intergroup analysis, as appropriate. The Spearman test was used for calculating the correlation between sPAP and Eng levels. Two standard deviations (SD) above mean were taken for calculating positive Eng values. *P* < .05 was considered significant for all analysis.

## 3. Results

The study included 60 subjects, 40 having a diagnosis of SSc and 20 healthy control subjects (20 SSc-sPAP patients and 20 SSc-non sPAP patients). In the SSc group, 26 patients presented lSSc and 14 patients dSSc; 28 were female and 12 were men, having an average age of 44.3 ± 9.8 at disease onset. 

Disease duration was 8.57 ± 5.2 years, measured from the point at which the first symptom appeared. The time from the onset of Raynaud's phenomenon was 9.5 ± 5.9 years and Rodnan's score [15] was 21.7 ± 10.2. All patients presented telangiectasias; 12 (30%) patients had calcinosis, 28 (70%) patients had gastrointestinal involvement, and mean sPAP value was 57.75 ± 14.5 mmHg in SSc-sPAP patients. Sixteen patients from the SSc-sPAP group (16/20) were diagnosed with > 35 mmHg sPAP and >3 m/second tricuspid regurgitation velocity; only four (4/20) had >3 m/second tricuspid regurgitation velocity. Sixteen SSc-sPAP patients (80%) had >48 mmHg sPAP and four (20%) had <48 mmHg sPAP but more than 40 mmHg in sPAP and had no significant structural right atrial and/or ventricular damage. Forty-five percent had calcinosis in the SSc-sPAP group, whereas only 15% were present in SSc-non sPAP group (*P* = .041).

HRCTT revealed no ILD-related findings in any patient. No abnormality was found in 12 patients (60%) and PAH findings such as dilatation of proximal and segmental pulmonary arteries were found in 8 (40%). No patients had bilateral reticular linear or reticulonodular densities in the lung bases or opacities reticular, ground-glass opacities, or honeycombing in HRCTT. 

Anticentromere antibodies (ACA) were reported to be positive in 27 patients (67.5%) and anti-Scl-70 in 9 patients (22.5%). Seventy-five percent had positive ACA in the SSc-sPAP group (5/7 having diffuse SSc and 10/13 having limited SSc), whereas antibodies anti-Scl-70 were positive in 10% of that group (2/7 SSc-sPAP patients and diffuse SSc). A speckled antinuclear antibody (ANA) pattern was present in 15% (3/13 SSc-sPAP patients and limited SSc). No patient presented renal crisis or ischemic cutaneous ulcers, but all presented telangiectasias. Table 1 gives the clinical characteristics for the SSc patients.

**Figure 1:**
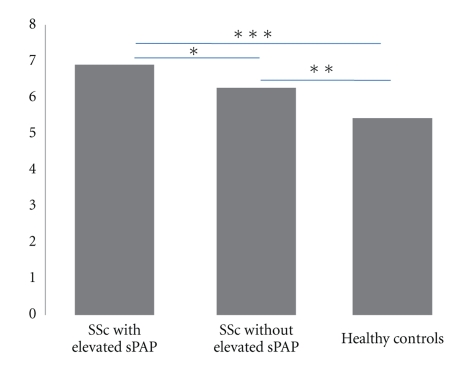
Eng levels in SSc with elevated sPAP, without elevated sPAP, and in healthy controls. Serum levels of Eng in patients with SSc and elevated sPAP, SSc without elevated sPAP, and healthy control subjects (mean ± SE). Mean serum levels were statistically higher in the SSc group compared to the control group (SSc group *cf* healthy controls *P* = .0028; SSc with elevated sPAP *cf* SSc without elevated sPAP *P = .2447; SSc without elevated sPAP *cf* healthy controls **P = .057; SSc with elevated sPAP *cf* healthy controls ***P = .0006).

Serum levels of Eng in SSc-sPAP patients tended to be higher than in SSc-non sPAP patients, having no significant statistical difference (*P* = .2447) and were higher than healthy controls (*P* = .0006). Mean values were 6.89 ng/dL, 6.20 ng/dL, and 5.42 ng/dL and median values were 7.07 ng/dL, 6.01 ng/dL, and 5.42 ng/dL, respectively (Figure 1).

There was no difference between the SSc-non sPAP group and healthy controls (*P* = .057). Intergroup analysis revealed a difference between the three groups (Kruskal-Wallis test, *P* = .0037) which was mainly due to SSc-sPAP group compared to the healthy control group. Categorized analysis of serum levels from Eng reported a difference between all groups (*P* = .003); however, detailed analysis revealed interesting findings. The SSc-sPAP group consisted of eleven patients having positive Eng (OR = 2.85; 0.65–12.88 95% CI; *P* = .11, when was compared to the SSc-non sPAP group); the SSc-non sPAP group had six patients (OR = 8.14; 0.8–393.74 95% CI; *P* = .046, when compared to healthy controls) and one healthy control (OR = 23.22; 2.46–1050.33 95% CI; *P* = .001 when was compared to the SSc-PAH group). An association was reported for positive Eng (OR = 14.04; 1.79–617.05 95% CI; *P* = .0028) when SSc-sPAP and SSc-non sPAP groups were pooled and compared to healthy controls. There was no correlation between Eng levels and sPAP (Rho = 0.1384, *P* = .39).

## 4. Discussion

Two clinical hallmarks for SSc are its clinical heterogeneity and the wide range of vascular and fibrotic manifestations. Organ involvement, different patterns, and internal organ manifestation severity are the global outcome's most significant determinants [16].

PAH is the main cause of morbidity and mortality amongst vascular complications for SSc. A diagnosis of PAH has occurred late in the course of the disease until now and right heart catheterization has been the gold standard for its diagnosis. However, this diagnosis test is invasive and implies morbidity and mortality risks. The echocardiogram is a noninvasive technique and its limitations include it being operator-dependent and its false positive rate is close to 30% [16].

An imbalance in circulating angiogenic factors in SSc may be associated with vascular endothelial dysfunction. Eng is one of the factors supporting vascular integrity, with this being a 180 kDa homodimeric coreceptor for TGF-*β* superfamily members which is predominantly expressed on endothelial cell surfaces [17].

Eng may have roles in hematopoiesis, cardiovascular development, and angiogenesis and is highly expressed on vascular endothelial cells [18], chondrocytes [19], and term placenta syncytiotrophoblasts [20]. It is also found on monocytes [21], erythroid precursors [22], and a hematopoietic stem cell subpopulation [23]. Although its role remains elusive, circulating soluble Eng levels are raised in patients suffering from atherosclerosis [24] and certain cancers including breast [25], colon [26], and myeloid malignancies [27]. Eng is likely to be involved with angiogenesis in endothelial cells, since prominent Eng expression has been demonstrated in neovascular states, including the enhanced vascularity of psoriasis [28].

Previous reports have shown that the Eng gene is located at 9q34.1 and that mutations of this gene having reduced Eng expression are responsible for one of the two types of hereditary hemorrhagic telangiectasia, an autosomal dominant disorder characterized by multiple telangiectasia of the skin, mucous membranes, gastrointestinal tract, arteriovenous malformation, and pulmonary hypertension [29]. 

Leask et al. [6] found that the endothelial-enriched high-affinity TGF-*β* receptor endoglin was up-regulated in dermis fibroblasts cultured from involved areas of skin taken from SSc patients related to normal fibroblasts and that Eng expression increased with the disease's progression, suggesting that Eng might represent a potential marker for staging SSc. Another finding was that Eng overexpression in fibroblasts blocked the accumulation of activated nuclear Smads and suppressed TGF-*β* ability to induce connective tissue growth factor (CTGF) profibrotic cytokine target gene promoter. Such results suggest that SSc fibroblasts induce Eng expression to suppress TGF-*β* induction of gene expression in a negative feedback loop.

Fujimoto et al. [30] examined soluble Eng serum levels in SSc patients and found these levels to be higher in patients having lSSc, telangiectasias, and ACA. Furthermore, SPPA was positively correlated with Eng levels in patients having lcSSc, but only two of these patients presented PAH. There was no difference in heart, esophageal, or renal involvement between patients having higher Eng serum levels and those having normal levels.

Wipff et al. [17] demonstrated an association between Eng gene polymorphism and PAH, and another study by the same authors [31] found that Eng appeared to be increased in SSc and to be particularly associated with the vascular phenotype. They also showed a higher Eng concentration in SSc patients compared to healthy controls; they included 17 PAH patients and found that Eng serum levels in SSc patients with PAH were similar to those of patients without PAH. Higher Eng levels were found in SSc patients compared to healthy controls in a previous study by the present group [32], having a statistical difference between the two groups in favor of SSc and PAH patients. However, a correlation with sPAP value was not found, with a limitation of this study being the lack the comparison with patients with SSc without PAH.

Higher Eng levels were found in the two types of SSc in the current study, contrasting with the results of Fujimoto et al. [30]. The 20 patients in the current study presented elevated sPAP secondary to SSc, and Eng levels in these were more raised than in controls (SSc-non sPAP group and healthy controls). However, there was no statistical difference when the SSc-sPAP group was compared to the SSc-non sPAP group. These results differed from the recent ones reported by Wipff et al. [17] and suggest that Eng serum levels are elevated in SSc patients and not exclusively due to PAH. Endothelial activity in patients in the early stages of PAH may be a potential reason for finding elevated Eng serum levels. The difference in calcinosis percentage between the groups is striking, especially since calcinosis may develop in areas of poor perfusion and be the result of vascular disease [33].

Another interesting finding in this study was the presence of ACA in diffuse SSc. ACA have been associated with limited SSc and anti-topoisomerase I with diffuse progressive disease; however, 10 to 15 percent of patients with diffuse disease have these antibodies [34]. Coral et al. [35] reported positive ACA in 93% of patients studied in Colombia, independent of SSc subtype. 

It thus seems that the higher presence of positive ACA could be related to PAH; in fact earlier reports have associated these antibodies with increased risk of pulmonary hypertension [36, 37]. Telangiectasias were present in all patients in the present study; it had already been reported in Colombian SSc patients [35].

This work had the following limitations. Right heart catheretization (RHC) is now considered the gold standard for PAH diagnosis due to the presence of high false positive results with echocardiogram and also because RHC allows therapeutic choices for vasoreactivity seen in PAH to be evaluated. Elevated sPAP was assessed and diagnosed with echocardiogram in this work due to the Colombian Health System's limitations regarding performing RHC. There was a high correlation between the measurements obtained using both techniques when the cardiologist operator had broad experience in evaluating pulmonary arterial pressure and there was no change in right heart anatomical structural. Besides, >48 mmHg sPAP associated with >3.0 TRV was used for diagnosing moderate/severe PAH [38], thereby reducing the possibility of false positives occurring. Since our cohort had been continuously followed up, the cases were chosen during the early phases of PAH development to exclude even the smallest structural damage that could have altered echocardiographic evaluation. 

A second limiting concerned the fact that a relative small population was included in each branch to include biomarkers having a potentially pathophysiologic role for generating elevated sPAP and because not enough scientific papers have assessed it, meaning that the current paper can be considered to be a pilot-study.

## 5. Conclusion

Raised Eng serum levels were found in SSc patients (SSc-sPAP patients and SSc-non sPAP) compared to healthy controls, suggesting a role for Eng in SSc vasculopathy and not just in PAH. However, prospective studies are needed which include a larger population to verify these observations. Serum biomarkers could detect an early stage of the disease in patients having a high risk of developing elevated sPAP which could afford a better outcome during these patients' follow up.

## Figures and Tables

**Table 1:** SSc patients' clinical characteristics.

Clinical characteristics	SSc- sPAP patients	SSc-non sPAP patients	*P*
	*n* = 20	*n* = 20	
Age (in years)	54.4 ± 11	51.1 ± 13.7	.4
Age at onset	44.75 ± 10	43.9 ± 9	.78
Male : female	2.3 : 1	2.3 : 1	1
Duration (means ± SD), years	9.65 ± 4	7.5 ± 6.3	.19
lSSc	13	13	1
dSSc	7	7	1
sPAP mmHg at rest	57.75 ± 14.5	19.4 ± 12	< .0001
Raynaud%	100	100	1
Time of onset of Raynaud	9.1 ± 4	9.95 ± 6	.81
Rodnan score	22.1 ± 9	21.3 ± 10	.21
Calcinosis%	45	15	.041
Telangiectasias%	100	100	1
Renal crisis%	0	0	
Ischemic cutaneous ulcers%	0	0	
Gastrointestinal involvement%	60	80	.15
normal HRCTT%	60	100	NS
HRCTT PAH%	40	0	NS
Anticentromere ab%	75	60	.21
Anti*-*Scl*-*70 ab%	10	20	NS
ANA%	15	20	NS
Methotrexate	11	11	1
Cyclophosphamide	6	7	.73

SD: standard deviation, lSSc: limited systemic sclerosis, dSSc: diffuse systemic sclerosis, sPAP: systolic pulmonary arterial pressure, HRCTT: high-resolution computed tomography of thorax, PAH: pulmonary arterial hypertension. Fisher's or Wilcoxon test was used for calculating the differences between groups.
